# Effects of hay provision and presentation on cognitive development in dairy calves

**DOI:** 10.1371/journal.pone.0238038

**Published:** 2020-09-01

**Authors:** Kelsey C. Horvath, Emily K. Miller-Cushon

**Affiliations:** Department of Animal Sciences, University of Florida, Gainesville, Florida, United States of America; none, UNITED STATES

## Abstract

In the dairy industry, feeding management has considerable influence on calf behavioral development, yet there is limited understanding of how aspects of diet or accommodating more varied feeding behavior may affect cognitive development in young calves. The objective of this study was to evaluate effects of provision and presentation of hay on the cognitive ability of pre-weaned dairy calves. Individually-housed Holstein heifer calves were assigned at birth to 1 of 3 treatments: pelleted starter only (n = 10), hay (chopped to 5 cm) and starter provided in separate buckets (n = 12), or hay and starter offered as a mixture (n = 11). During week 5 of age, calves were tested daily in a learning task consisting of a T-maze with a milk reward (0.2 L milk) placed in one arm. Calves were subjected to an initial learning and reversal learning stage, where the reward location was changed to the opposite arm of the maze. Calves received 5 sessions/d until they met learning criterion (moving directly to correct side in 3 consecutive sessions) for initial and reversal learning. Dietary treatment did not affect pass rate or the number of sessions required to pass the initial learning stage. During the reversal learning stage, calves provided only starter had a lower pass rate (0.038, during first 8 testing session) early during testing than calves provided hay separately (0.20; *P* = 0.020) and tended to have a lower pass rate than calves provided hay as a mixture (0.14; *P* = 0.057). Calves provided only starter also tended to require more sessions to meet the learning criterion (15.8) than both calves provided hay separately (10.8; *P* = 0.089) and as a mixture (11.8; *P* = 0.10). Calves provided hay also kicked less and spent more time sniffing or licking the testing area. The results of this experiment indicate that provision of hay may affect behavioral flexibility in dairy calves.

## Introduction

Evidence across species points to an important role of early experience on behavioral development and cognition. In the dairy industry, calves are separated from the dam at birth, such that all aspects of housing and feeding are under human control and have considerable implications for behavioral development and cognition. For example, housing calves in social groups, compared to the industry norm of individual housing at birth, is known to broadly affect behavioral development and performance (reviewed by [1]). Dairy calves housed individually from birth also had impaired ability in a reversal learning task compared to calves housed with social contact [2,3], were more reactive in a novel environment [4], and did not habituate to a novel object over repeated testing [2]. In addition to aspects of social housing, the method of feeding young dairy calves has short and long-term influences on behavior and dietary learning [5] and feeding management factors which accommodate a broader range of natural feeding behavior may similarly influence cognitive development.

Whereas dairy calves are typically provided a single, uniform pelleted diet, provision of forage accommodates more varied feeding behavior and increases feeding time [6]. Dairy calves are motivated to access forage prior to weaning [7,8] and access to hay also reduces non-nutritive oral behaviors in individually housed calves [9,10]. This suggests that access to hay may be broadly beneficial for calves and support development of foraging related behaviors. Recent findings suggested that dairy calves provided with both hay and the opportunity to suckle milk through a teat, feeding management factors which accommodate a broader range of natural feeding behavior, had improved ability in a reversal learning task, compared to calves provided only a pelleted starter diet and milk from a bucket which prevents sucking behavior [11]. These previous findings indicate a role of feeding management in cognitive development.

Supporting early cognitive development in dairy calves may have important applied significance, as the ability of dairy cattle to adapt to management changes they routinely encounter as they develop may depend on learning ability or behavioral flexibility. Behavioral flexibility refers to an individual’s ability to adjust their behavior in response to changing environmental cues [12] and is often assessed using a reversal learning task, where the ability to relearn an associative task is measured [13]. In dairy calves, previous evidence suggests that individual ability in a reversal learning task was associated with increased exploratory behavior and reduced latency to begin feeding after movement to a novel housing environment [14]. While these results describe a correlation only, it is possible that factors which improve calf performance in a reversal learning task may have consequences for their subsequent ability to cope with environmental changes.

To further evaluate the role of dietary factors in cognitive development in dairy calves, the objective of this study was to examine the effects of hay provision alone, either in a separate feeding location from a conventional pelleted calf starter or as a single diet mixed with starter, on the ability of calves to learn and relearn the location of a reward in a T-maze. We hypothesized that, if provision of forage and opportunity for more varied feeding behavior early in life improves behavioral flexibility, then calves that received hay would have improved success during the reversal learning task. We were further interested in the method of providing hay, given that cattle evolved selecting from spatially distributed feed sources [15] and foraging strategies across species depend on spatial learning and memory [16]. We speculated that opportunities for foraging in multiple feeding locations may contribute to possible benefits for cognitive development, and therefore hypothesized that provision of hay separately from starter may improve success during the cognitive task.

We measured the ability of calves to perform an initial and reversal learning task conducted in a T-maze by measuring pass rate by session and the number of sessions required to meet the learning criterion. Evidence across species also suggests that success in initial and reversal learning tasks may also coincide with behavioral differences within the testing arena [11,17]. For example, in previous work we observed effects of feeding management on duration of time in the incorrect arm and frequency of kicking within the maze, possibly indicating differences in reactivity to the testing area [11]. Therefore, we characterized behavior of calves within the test during the reversal learning stage, to evaluate whether possible differences in reversal learning ability coincided with other differences in behavioral responses to testing. Finally, to evaluate the validity of our assumption that the reversal learning stage of testing was an indicator of behavioral flexibility distinct from initial learning ability, we analyzed the association between sessions to pass the initial and reversal learning stages.

## Materials and methods

### Animals and housing

Thirty-three Holstein heifer calves were enrolled at birth into the study at the University of Florida Dairy Unit (Hague, FL). All study procedures were reviewed and approved by the University of Florida Animal Care and Use Committee (IACUC Study #201609416). Calves were reared in individual wire-mesh pens (0.9 × 1.8 m; width × depth) that permitted visual and auditory, but no tactile contact with other calves according to the standard operating procedures for this facility. They received 4L of quality-controlled colostrum and were uniquely identified with RFID ear tags. They received pasteurized waste milk mixed with a powdered enhancer (Pasteurized Milk Balancer Protein-Blend, Purina Animal Nutrition LLC, Shoreview, MN) delivered at 0600h and 1800h in a teat bucket. All calves received 6 L/d in two meals for the first two weeks of life followed by 8 L/d until weaning began at 49 days of age. During the week of cognitive testing (as described below; week 5 of life), calves received 1 L of milk during the test as a reward, so 1 L of milk was removed from the second meal to maintain a total of 8 L/day. All calves had *ad libitum* access to solid feed, presented according to dietary treatments described below, and water. As per standard operating procedure at the University of Florida Dairy Unit, all calves were disbudded by a University of Florida veterinarian during week 4 of life by hot iron and provided both local anesthesia and analgesia during the procedure.

### Experimental design

Calves were randomly assigned to one of three treatments from birth (randomization conducted using the random group number generator in Microsoft Office Excel, Microsoft, Redmond, WA), differing in provision and presentation of hay: starter only (ST; n = 10), hay provided separately from starter (SEP; n = 12), or hay provided as a mixture with starter (MIX; n = 11). We enrolled calves to target 8 calves/treatment during the reversal learning stage (based on previous data describing effects of feeding method and social housing on cognitive test outcomes [2,11]) with additional calves enrolled to account for potential failure during initial stages of testing. The starter ration was a pelleted diet (22.8% CP and 26.6% NDF; Ampli-Calf Starter 20 Warm Weather, Purina Animal Nutrition LLC, Shoreview, MN, USA) and the hay was chopped (5 cm) coastal Bermuda grass (9.6% CP and 74.8% NDF). Calves that received hay mixed with starter were provided their diet in a single bucket. The mixed diet consisted of 80% starter and 20% hay (on a dry matter basis), and the calves that received hay separately were offered the same ratio of feed components.

Solid feed intake was measured during the week of testing, as differences in feed intake may affect motivation for the milk reward. Feed intake (on a dry matter basis) of solid feed during the week of cognitive testing (week 5; described below) was determined by weighing offered and refused feed daily, with pooled samples of fresh and orts samples oven-dried within 4 hours of collection for 48 h at 55°C to determine dry matter content and calculate feed intake on a dry matter basis.

### Cognitive test

**Fig 1:**
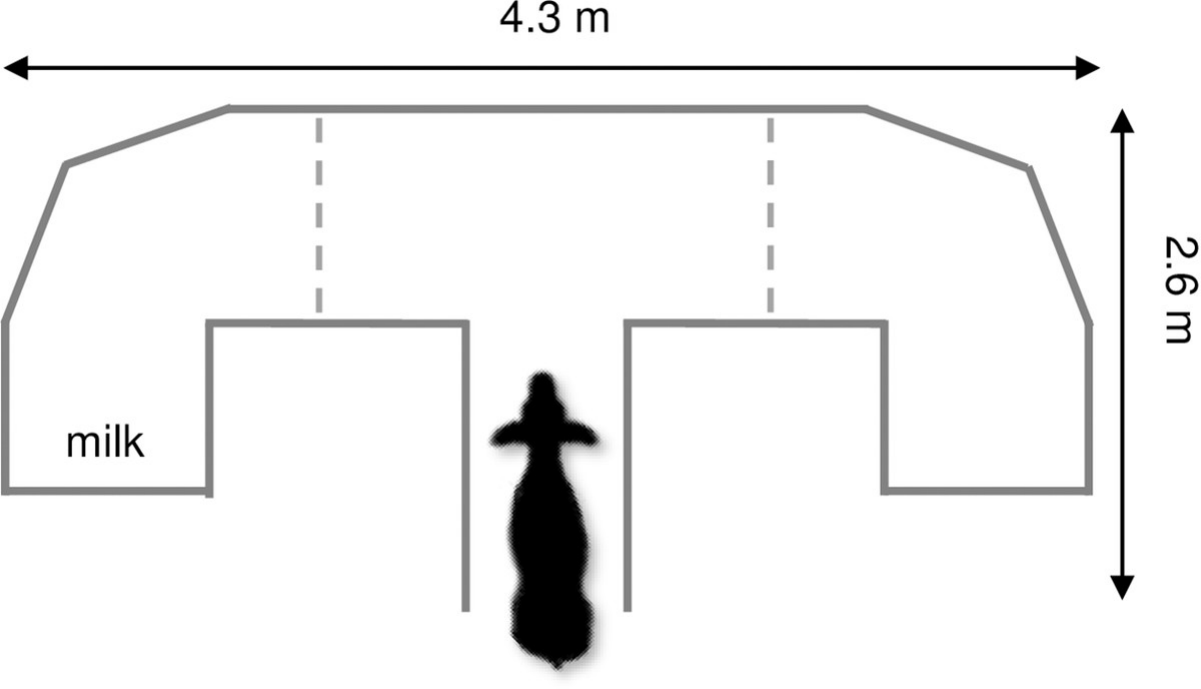
Diagram of the T-test used to conduct the cognitive test. The milk reward was located in the marked area, with initial side randomized between calves. Dashed lines indicate division of the maze into different areas (correct or incorrect arm, depending on location of reward, and middle).

During week 5 of life, cognition was assessed using a learning task in a T-maze (Fig 1). The test was modeled after a similar study performed in pigs [18], which was previously adapted for use in dairy calves [11]. The maze was constructed using 24 panels (0.61 x 1.22 m) of reconfigurable wire mesh pens (Oxgord Dog Animal Large Metal Wire Playpen 48 inches, OxGord Inc., Los Angeles, CA, USA). The wire mesh panels were covered with corrugated plastic to provide a visual barrier to both the reward and to the environment outside of the maze. The maze was supported on the outside by stacked bales of straw. Calves entered into the maze through the base of the “T” and exited through the arm containing the reward, which was allowed to open after the calves found their reward.

Each calf received a maximum of 5 sessions per day for 5 days or until criterion (moving directly to and begin consuming the reward in 3 consecutive sessions) was reached for both learning tasks. An additional session was provided if the calf was only one session away from meeting the learning criterion. Each session had a maximum time of 3 minutes to complete the task. A reward of 0.2 L milk was placed in one arm of the maze and was balanced between each side for each treatment to prevent an effect of laterality [19]. Open containers of milk were placed outside each arm of the maze and out of sight of the calves to prevent calves from using olfactory cues to find the reward. If any of the milk reward allocation remained at the end of testing each day (e.g. due to repeated failure to find the reward, or due to early completion of the stage of testing), calves were allowed to finish the remaining milk before returning to their home pen, such that milk intake was consistent across all calves. On the first session on the initial learning stage, each calf was guided to the correct arm and allowed to drink the milk reward. To guide the calf, a member of the study staff entered the maze behind the (un-haltered) calf and allowed the calf to move freely, only extending their arm to block movement towards the incorrect arm or gently applying pressure on the calf’s rump if they did not move voluntarily, relieving the pressure when the calf took a step.

Calves were allowed 11 total sessions to meet criterion for initial learning, and calves unable to meet criterion were considered to not learn the task and were removed from further testing. The fewest sessions any individual calf completed was 3 to meet the learning criterion, and calves were removed from further testing if they did not complete initial learning by the second day of testing (with a maximum of 10, or 11 sessions if the calf was only one session away from meeting the learning criterion on the last day).

Calves that met the learning criterion for the initial learning stage (moving directly to and begin consuming the reward in 3 consecutive sessions), were tested in a reversal learning task where the location of the reward was changed to the opposite arm. Calves were tested from completion of initial learning until the fifth consecutive day of testing. The fewest sessions any individual calf completed was 6 to meet criterion, and the slowest learner required 21 sessions to meet the reversal learning criterion.

The behavior of all calves was recorded continuously during each session by video camera (GoPro Hero3 and GoPro Hero7, GoPro Inc., San Mateo, CA, USA). The video was reviewed using Behavioral Observation Research Interactive Software [20] to measure additional behaviors as defined in Table 1 for the first 5 sessions of reversal learning. Behavior was recorded from video by a single, trained observer blind to treatments, and intra-observer reliability was calculated with Pearson correlation coefficients > 88% for all behaviors observed.

**Table 1:** Ethogram of behaviors observed from video recorded during the first five sessions of reversal learning.

Behavior	Description
Total time	Duration of time from entering maze until beginning milk consumption
Middle duration	Duration spent in middle zone of maze
Correct duration	Duration spent in the arm with the reward
Incorrect duration	Duration spent in the arm without the reward
Kick/buck	Number of times legs were lifted from ground higher than a normal step
Licking/sniffing	Duration with mouth less than 5 cm from parts of maze other than reward

### Statistical analysis

Statistical analyses were performed using SAS v. 9.4 (SAS Institute Inc., Cary, NC, USA) with calf as the experimental unit. A total of 33 calves (n = 10 for ST, n = 11 for MIX, and n = 12 for SEP) were included in analysis for the initial learning stage, and a total of 26 calves (n = 7 for ST, n = 10 for MIX, and n = 9 for SEP) were included in the analysis for reversal learning stage, as not all calves met the learning criterion for the initial learning stage.

Given evidence in the psychology literature that enrichment affects success during early sessions of testing in both initial and reversal learning tasks [17], we analyzed pass rate during the first testing sessions for both stages. The cut-off for sessions for inclusion in this analysis was set at the 25% percentile for sessions required to meet the learning criterion in each stage (first 5 sessions of initial learning and the first 8 sessions of reversal learning), as calves were excluded from subsequent test sessions as they met the learning criterion and inclusion of sessions beyond this point yielded missing values for an increasingly large fraction of calves. This analysis of outcome by session was analyzed separately by stage (initial or reversal learning) using generalized estimating equations within Proc Genmod, which are designed for analysis of data with correlated errors, as is the case with longitudinal data sets. The model included effects of treatment, session, and side (L or R) of the reward, with calf as the subject for repeated measures across sessions (modeled with an exchangeable working correlation structure).

To determine effects of treatment on overall success during each stage of testing, the total number of test sessions required to meet the learning criterion were analyzed separately by stage (initial or reversal learning) in a general linear mixed model (Proc Glimmix), including treatment and side (L or R) of the reward as fixed effects. Training was not continued beyond 11 sessions for the initial learning stage and 21 sessions for the reversal learning stage. Therefore, any calves that had not reached the learning criterion for that stage (passing 3 consecutive sessions) were assigned the value of 12 session for the initial learning stage and 22 sessions for the reversal learning stage. The number of test sessions to meet the learning criterion were log-transformed to meet assumptions of normality, and variances were estimated separately by treatment to account for unequal variances. Ability to meet the learning criterion (pass/fail) for both stages (initial and reversal learning) was analyzed in a similar model, but fit with a binary distribution.

To evaluate whether success during the initial learning stage was predictive of success during the reversal learning stage, we performed a regression between the number of sessions to meet the learning criterion for the initial and reversal learning stages (fit using a negative binomial distribution in Proc Genmod). This analysis was conducted across all calves that passed the initial learning stage and were tested in the reversal learning stage (n = 26), and repeated by treatment to evaluate whether correlation between performance in both stages of testing varied by treatment.

Behavioral data during the first 5 sessions of reversal learning was missing for 3 calves due to video failure and was therefore analyzed for n = 8 calves on MIX treatment, n = 6 calves on ST treatment, and n = 9 calves on SEP treatment. Duration of time in each area of the maze was analyzed in Proc Glimmix, with effects of treatment and reward side and repeated data across sessions modeled with a compound symmetry covariance structure with calf as subject. These variables were log-transformed to meet assumptions of normality (as screened using the Univariate procedure of SAS). Duration of time spent licking or sniffing the maze and frequency of kicking in the maze were transformed using the two-parameter Box-Cox transformation, where g(y;λ1,λ2) = log(y+λ2) with λ1 = 0 and λ2 = 1 which accommodated the presence of zero values in the data set.

Solid feed intake during the week of testing was averaged across days and analyzed in Proc Mixed with treatment as a fixed effect and calf as a random effect.

Model residuals were examined to confirm assumptions of homogeneity of variance. Significance was declared at *P* < 0.05, with trends reported if 0.05 ≤ *P* ≤ 0.10. Results are reported as least square means (back-transformed, in the case of data transformed to meet assumptions of normality) with 95% confidence intervals in brackets.

## Results

**Fig 2:**
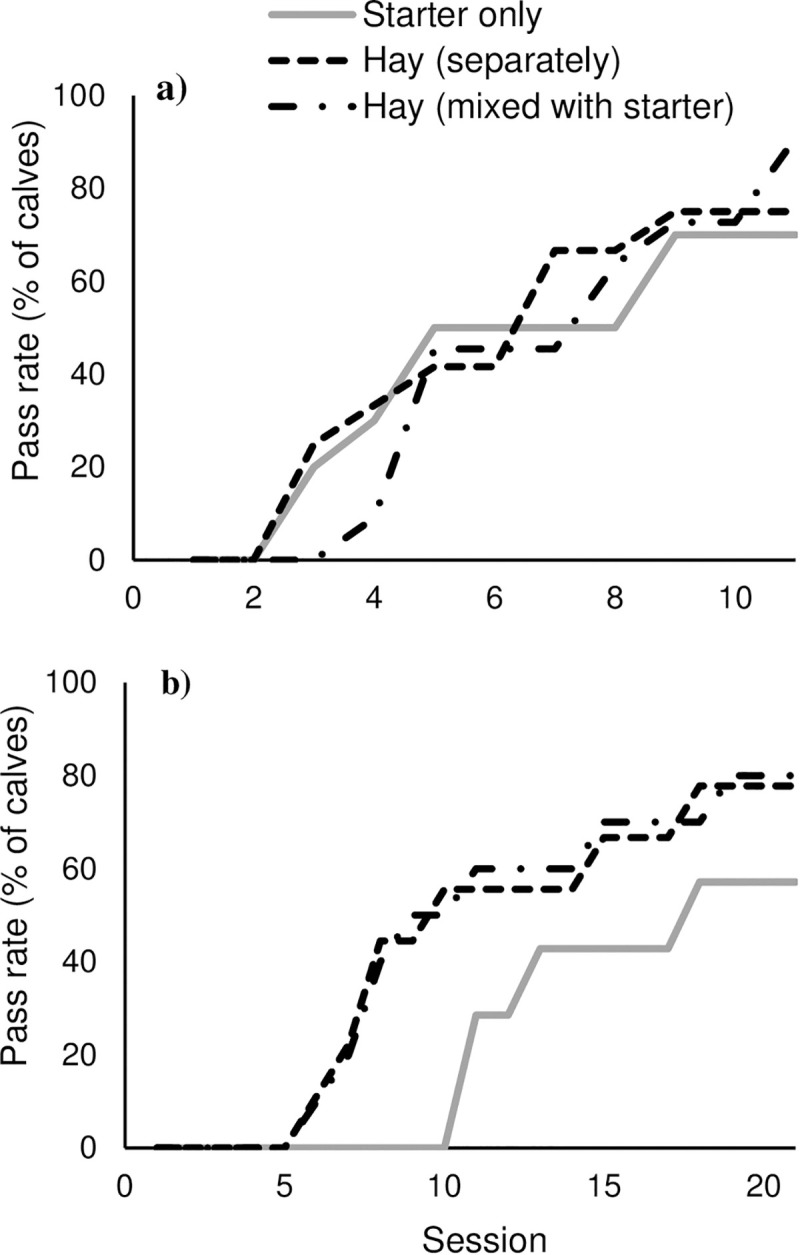
The percentage of calves meeting the learning criterion by session of testing for (a) initial learning and (b) reversal learning.

During the initial learning stage of the test, probability of passing did not differ between treatments during the first 5 test sessions [ST: 0.53 (0.28, 0.77); MIX: 0.37 (0.19, 0.59); SEP: 0.43 (0.21, 0.67); Z > -0.91; *P* > 0.36]. Overall, success during the initial learning stage did not differ between treatments, either in the number of sessions required to meet the learning criterion [ST: 6.5 (4.5, 9.3); SEP: 6.1 (4.5, 8.3); MIX: 6.7 (5.3, 8.4); F_2,29_ = 0.14; *P* = 0.87; Fig 2A] or overall pass rate [70% vs. 75% vs. 91%; ST vs. SEP vs. MIX; F_2,29_ = 0.63; *P* = 0.54]. Calves that passed the initial learning stage (n = 7 for ST, n = 10 for MIX, n = 9 for SEP) proceeded to the reversal learning stage.

During the reversal learning stage, calves provided only starter had a lower pass rate early during testing [0.038 (0.012, 0.12); assessed during the first 8 sessions of testing] than calves provided hay separately [0.20 (0.082, 0.42); Z = 2.33; *P* = 0.020] and tended to have a lower pass rate than calves provided hay as a mixture [0.14 (0.047, 0.36); Z = 1.9; *P* = 0.057]. This is reflected in Fig 2B which shows a lower percentage of ST calves meeting the learning criterion earlier during the reversal learning stage. Calves provided only starter also tended to require more sessions to meet the learning criterion 15.8 (12.5, 20.1) than both calves provided hay separately [10.8 (7.4, 15.8); t_22_ = -1.78; *P* = 0.089] and as a mixture [11.8 (9.0, 15.4); t_22_ = -1.71; *P* = 0.10]. The pass rate for calves tested in the reversal learning stage was 57% for ST, 80% for MIX, and 78% for SEP (F_2,22_ = 0.37; *P* = 0.70).

The number of sessions to meet the learning criterion during the reversal learning stage was not associated with sessions to meet criterion in the initial learning stage [across all calves: estimate = 0.0008 (-0.066, 0.068); *P* = 0.98]. Similarly, there was no association evident when this regression was performed separately by treatment (*P* > 0.70).

Behavioral observations during the first 5 sessions of the reversal learning stage (Table 2) revealed that calves that received hay separately from starter spent longer in the maze, and specifically spent more time in the middle section of the maze, than calves provided only starter, with calves provided hay as a mixture having intermediate responses. Duration of time in the correct and incorrect arm of the maze were not affected by treatment, but duration of time in each region of the maze decreased across sessions. Calves provided hay in any form kicked less frequently than calves provided starter only and spent more time licking or sniffing the maze during testing.

**Table 2:** Behavior of calves provided different dietary treatments: Starter only (ST), hay separately from starter (SEP), or hay mixed with starter (MIX) during the first 5 sessions (S) of the reversal learning stage in a T-maze. Data are reported as back-transformed means with 95% confidence intervals in brackets^1^.

	Treatment			Session		Treatment	
Variable	ST	MIX	SEP	F^2^	*P*	F^2^	*P*
Total time to find reward (s)	19.1^a^	24.1^a^^b^	40.4^b^	18.2	< 0.001	3.6	0.043
	(12.2, 29.9)	(16.5, 35.2)	(27.0, 60.6)				
Time in correct arm (s)	6.4	6.0	5.7	3.7	0.008	0.1	0.90
	(4.3, 9.3)	(4.2, 8.3)	(4.1, 7.9)				
Time in incorrect arm (s)	8.1	10.4	9.7	13.6	< 0.001	0.4	0.66
	(5.0, 12.6)	(7.0, 15.3)	(6.6, 14.0)				
Time in middle (s)	2.7^a^	5.4^a^^b^	11.8^b^	8.6	< 0.001	4.5	0.026
	(1.2, 6.1)	(2.7, 10.8)	(6.1, 23.0)				
Frequency of kicks (no/test)	2.9^a^	1.1^b^	0.58^b^	-	-	7.7	0.004
	(1.9, 3.9)	(0.2, 2.0)	(0, 1.4)				
Pen-directed licking/sniffing (s)	1.0^a^	3.4^b^	3.2^b^	-	-	2.9	0.078
	(0.2, 2.5)	(1.7, 6.1)	(1.6, 5.7)				

^1^Due to video recording failure, data were missing for 3 calves and analyzed for n = 8 calves on MIX treatment, n = 6 calves on ST treatment, and n = 9 calves on SEP treatment.

^2^For effect of treatments, df = 2,22 for total time to find the reward and df = 2,19 for all other variables (due to missing video data for 3 calves). For effect of session, df = 4,100 for total time to find the reward and df = 4,80 for other variables.

^a,b^Superscripts indicate significant differences (*P* < 0.05) between treatments.

Solid feed dry matter intake did not differ between treatments during the week of testing [mean with 95% confidence intervals in brackets: ST: 113.2 (69.6, 156.9); MIX: 110.4 (68.8, 152.0); SEP: 106.3 (66.4, 146.1) g/d; F_2,30_ = 1.1; *P* = 0.66] and all calves finished their daily milk allotment (8 L/d, including milk provided during testing).

## Discussion

Consistent with our prediction, calves that were provided with hay passed the reversal learning stage sooner than calves that did not have hay. Ability to relearn a task is considered an indicator of behavioral flexibility [13], suggesting that calves that received hay may have increased behavioral flexibility evidenced by their ability to make the switch to going to the opposite arm sooner than calves that did not receive hay. In both mice and rats, most errors occur during the beginning of reversal learning in a T-maze test, and animals living in less complex environments generally have decreased performance and increased perseverative errors compared to those living in more complex environments [17,21]. Similarly, dairy calves housed with social contact had fewer errors during a reversal learning task than calves housed individually, suggesting that dietary complexity may have some similar effects as social contact on cognition [2]. We encourage further research to evaluate potential additive effects of social housing and hay provision.

We further predicted that provision of hay in a separate location from the starter may have an additional benefit, compared to provision of hay and starter as a mixture, due to the opportunity of foraging in multiple locations. However, results in the present study indicated that hay provision in any form influenced cognition, with no distinct differences in outcomes between calves provided hay separately or as a mixture. It is important to note that calves provided mixed diets will often ‘sort’ within the diet, selectively consuming certain feed particles and avoiding others [7]. As such, it is possible that provision of feed types in separate locations may not have impaired the calf’s ability to choose between diets or forage in a more complex way. However, the specific effects of opportunity to forage in multiple locations on learning may be further investigated through provision of multiple sources of an identical feed type or varying the location of the feed bucket.

We did not see any effect of dietary treatment on performance during the initial learning stage, which is considered an indicator of general learning ability [13]. Response during the initial learning period did vary between individuals, in terms of sessions required to meet the learning criterion and overall pass rate, suggesting that factors other than early life experience may additionally influence individual learning ability. However, success during the initial learning stage was not predictive of ability to relearn the task during the reversal learning stage, with no correlation between testing stages in sessions required to meet the learning criterion. This suggests that the reversal learning stage was assessing a different aspect of cognition, such as behavioral flexibility [13]. Consistent with our findings, success during an initial stage of learning was not previously affected by social contact [2] or feeding method [11].

In previous work, we found that calves offered hay and milk through a teat spent less time in the incorrect arm of the maze during reversal learning, than calves that received no hay and were bucket fed milk [11]. Accordingly in the present study, we characterized behavior within the maze to shed light on potential behavioral differences that may affect success. In the present study, we found no effect of hay provision on duration of time spent on the incorrect side of the maze. Direct comparison of these results with previous findings may be limited due to differences in milk feeding method that were previously examined in combination with hay provision [11], where the duration of time spent on the incorrect arm of the maze may have been due to increased time spent interacting with the empty milk bucket. In the present study, calves provided hay actually took longer to find the reward during initial sessions of reversal learning, due to more time spent in the middle of the maze. In contrast, calves provided no hay were prone to ‘failing quickly’, where they went immediately to the incorrect arm before quickly moving to the correct arm, and they persisted in these perseverative errors for more sessions. Consistent with previous findings [11], we also found that calves provided hay also kicked less and spent more time licking or sniffing parts of the maze. These behavioral differences may suggest differences in exploration or reactivity associated with the change in reward location. Similar differences in behavior have been attributed to effects of social housing. For example, pair-housed calves spent more time exploring and kicked less than individually-housed calves during a social novelty test [4]. While the mechanism through which social contact affects reactivity to novelty may be different, increasing environmental complexity in general may affect calves’ exploration and response to a testing arena, which could affect learning.

In general, environmental complexity is understood to influence development of neural pathways related to learning and memory [22], which can help explain why animals living in physically and/or socially complex environments perform better in learning tasks than animals in impoverished environments [21,23]. While hay provision may seemingly contribute little in terms of environmental complexity, it provides opportunity for more varied behavior in calves reared in relatively restrictive environments. However, the specific mechanisms by which hay provision may affect reversal learning ability are unclear. Possible mechanisms may include aspects of sensory or olfactory stimulation, the physical action of chewing and ingesting hay, and digestive effects.

It is possible that feed variety in general may be stimulating for developing calves. Cattle have a sensitive ability to detect flavor [24], and there is evidence that varied feed choices may be preferred by young ruminants [25] and reduce stress compared to provision of a single uniform diet [26]. Feed variety may, then, reduce boredom and benefit calves by expanding the complexity of feed choice and foraging behavior available to them.

Specific sensory aspects of hay may also be critical. In naturalistic settings, calves begin consuming small amounts of forage from pasture within the first weeks of life [27], and evidence across species suggests possible benefits of exposure to preferred natural stimuli, including reduced stress (reviewed by [28]). Specifically, exposure to odors associated with plants (leaf-derived alcohols and aldehydes, or ‘green odours’) has been found to reduce physiological response to stressors and risk assessment behavior, suggesting reduced anxiety, in laboratory rodents (reviewed by [29]). While these effects have yet to be explored in cattle, it is well established that anxiety impacts cognition [30], and effects of natural stimuli on stress responsivity and cognition further research in livestock species.

In addition to sensory aspects of forage provision, the opportunity to chew and manipulate forage may affect cognitive development. Across species, there is a well-established link between mastication and cognitive ability (reviewed by [31]). While this has been well-studied in relation to age and cognitive decline, there is also evidence of a developmental effect of mastication. In rodents, impaired mastication during the first weeks of life (through provision of powdered diets) has been shown to impair spatial learning ability and memory in later testing [32,33]. Provision of pelleted diets to dairy calves in the absence of forage may similarly impair mastication; hay provision increases feeding time in calves [34], and similarly increasing dietary forage content increases chewing time in adult cattle [35].

Impaired mastication in animals not provided forage access may also relate to development of abnormal oral behaviors. Hay provision has been shown to reduce the duration of time calves will perform abnormal oral behaviors, such as pen-directed sucking [9,10]. More generally, living in less complex environments can cause more rigid behavioral repertoires to develop, including abnormal behaviors when an animal is unable to perform highly motivated behaviors due to limited complexity in the environment [36]. In other species, increased performance of abnormal behavior is associated with reduced behavioral flexibility; for example, this has been demonstrated in cribbing horses [37,38] and more extensively in laboratory animals performing abnormal repetitive behaviors (as reviewed by [39]). While abnormal oral behavior was not evaluated in calves in the present study, we encourage further research specifically examining associations between dietary factors, development of abnormal oral behaviors, and behavioral flexibility in cattle.

Finally, the digestive effects of hay in the diet may also play a role in behavioral and cognitive development. Provision of hay to dairy calves is known to affect the gut by buffering the rumen [40] and altering the gut microbiome [41,42]. In mice, increased gut microbial diversity reduces stress responses to physical restraint [43] and reduces protein expression used in the neural circuity for fear and anxiety [43,44]. Possible interactions between the gut and brain in cattle remain unexplored and warrant further investigation.

## Conclusions

In summary, we found that offering hay in any form improved the speed with which pre-weaned dairy calves were able to pass a reversal learning task. We also found that the form of presenting hay had varying effects on behavior in the testing arena, suggesting a need to further investigate effects of feed variety and presentation on calf behavioral development. While provision of hay may be viewed as a very minimal improvement to environmental complexity, our observed effects of diet on cognitive development in young calves underlines the need to accommodate much greater behavioral variety in these restrictive housing systems.
